# Retraction: Silicon-based induced resistance in maize against fall armyworm [*Spodoptera frugiperda* (Lepidoptera: Noctuidae)]

**DOI:** 10.1371/journal.pone.0273461

**Published:** 2022-08-31

**Authors:** The PLOS ONE Editors

The *PLOS ONE* Editors retract this article [1] because it was identified as one of a series of submissions for which we have concerns about authorship, competing interests, and peer review. We regret that the issues were not addressed prior to the article’s publication.

Additionally, after this article was published, it was noted that the names of Kexin Zhang and Changzhong Liu appear incorrectly in the author list. At the time of retraction, this article was republished to provide the correct names and citation.

IUH, CL, ATKZ, and AMA did not agree with the retraction. AK, RI, KZ, SA, and AAH either did not respond directly or could not be reached.
